# Greenhouse gas emissions, carbon stocks and wheat productivity following biochar, compost and vermicompost amendments: comparison of non-saline and salt-affected soils

**DOI:** 10.1038/s41598-024-56381-y

**Published:** 2024-04-02

**Authors:** Zia Ur Rahman Farooqi, Ayesha Abdul Qadir, Sehrish Khalid, Ghulam Murtaza, Muhammad Nadeem Ashraf, Shafeeq-ur-Rahman, Wasim Javed, Muhammad Ahmed Waqas, Minggang Xu

**Affiliations:** 1 Institute of Soil and Environmental Sciences, University of Agriculture, Faisalabad, 38040 Pakistan; 2 Water Science and Environmental Engineering Research Center, College of Chemical and Environmental Engineering, Shenzhen University, Shenzhen, 518060 China; 3 MOE Laboratory for Earth Surface Processes, College of Urban and Environmental Sciences, Peking University, Beijing, 100871 China; 4 Punjab Bioenergy Institute, University of Agriculture, Faisalabad, 38040 Pakistan; 5 Department of Agroecology, Aarhus University, Blichers Alle 20, PO BOX 50, 8830 Tjele, Denmark; 6 Institute of Eco-Environment and Industrial Technology, Shanxi Agricultural University, Shanxi Province Key Laboratory of Soil Environment and Nutrient Resources, Taiyuan, 030031 China

**Keywords:** Greenhouse gas emission, Wheat productivity, Global warming potential, SOC, Biochar, Compost, Biogeochemistry, Climate sciences, Environmental sciences

## Abstract

Understanding the impact of greenhouse gas (GHG) emissions and carbon stock is crucial for effective climate change assessment and agroecosystem management. However, little is known about the effects of organic amendments on GHG emissions and dynamic changes in carbon stocks in salt-affected soils. We conducted a pot experiment with four treatments including control (only fertilizers addition), biochar, vermicompost, and compost on non-saline and salt-affected soils, with the application on a carbon equivalent basis under wheat crop production. Our results revealed that the addition of vermicompost significantly increased soil organic carbon content by 18% in non-saline soil and 52% in salt-affected soil compared to the control leading to improvements in crop productivity i.e., plant dry biomass production by 57% in non-saline soil with vermicompost, while 56% with the same treatment in salt-affected soil. The grain yield was also noted 44 and 50% more with vermicompost treatment in non-saline and salt-affected soil, respectively. Chlorophyll contents were observed maximum with vermicompost in non-saline (24%), and salt-affected soils (22%) with same treatments. Photosynthetic rate (47% and 53%), stomatal conductance (60% and 12%), and relative water contents (38% and 27%) were also noted maximum with the same treatment in non-saline and salt-affected soils, respectively. However, the highest carbon dioxide emissions were observed in vermicompost- and compost-treated soils, leading to an increase in emissions of 46% in non-saline soil and 74% in salt-affected soil compared to the control. The compost treatment resulted in the highest nitrous oxide emissions, with an increase of 57% in non-saline soil and 62% in salt-affected soil compared to the control. In saline and non-saline soils treated with vermicompost, the global warming potential was recorded as 267% and 81% more than the control, respectively. All treatments, except biochar in non-saline soil, showed increased net GHG emissions due to organic amendment application. However, biochar reduced net emissions by 12% in non-saline soil. The application of organic amendments increased soil organic carbon content and crop yield in both non-saline and salt-affected soils. In conclusion, biochar is most effective among all tested organic amendments at increasing soil organic carbon content in both non-saline and salt-affected soils, which could have potential benefits for soil health and crop production.

## Introduction

According to the Intergovernmental Panel on Climate Change (IPCC), agriculture is among the primary sources of greenhouse gas (GHG) emissions^1^, contributing 10–14% of total GHG emissions, including 50–60% of the nitrous oxide (N_2_O) emitted^2^. Climate change associated with GHG emissions affects soil health, crop yield and biodiversity^3^. Soil organic matter (SOM) plays a key role in the global carbon cycle^4^. Soil organic carbon (SOC) stocks were reported by Lal^5^ to be 1550 Pg, which is two times greater than the carbon (C) in the atmosphere (760 Pg C) and nearly three times greater than that in vegetation (560 Pg C)^5^. Soil salinity and vegetation cover significantly affect SOC sequestration and net emissions of carbon dioxide (CO_2_) and other GHGs. Therefore, SOC stocks should be increased by managing soil cover and vegetation, and applying soil amendments^6,7^. Salt-affected soils have lost SOC due to poor management, and so have a high capacity to sequester C and a reduced capacity to emit GHGs^8^. A total area of 833 Mha soils are salt-affected worldwide. Of this, 85% are saline, 10% are sodic and 5% are saline-sodic^9^. In Pakistan, the area of salt-affected soils is 6.67 Mha^10^. Both natural (rocks weathering, precipitation and evaporation, geography) and human-induced processes (excessive use of fertilizers brackish water, higher temperatures) are responsible for soil salinization^11,12^. In salt-affected soils, plants experience osmotic stress due to high salinity levels, which makes it challenging for them to absorb water and nutrients^13^. Due to lower biomass synthesis and plant growth as a result, less organic matter is released into the soil as plant residue^14^. High salt concentrations may impede microbial activity in salt-affected soils, lowering the rate of SOM decomposition and/or the amount of organic matter that builds up in the soil^15^. Poor soil structure in salt-affected soils cause SOM loss due to leaching and erosion^16^.

Generally, emissions of GHG are lower in salt-affected soils because they have lower levels of SOM and decomposing organisms than non-saline soils. However, these soils can be managed to increase C sequestration and reduce net GHG emissions through soil organic amendments^17^.

With increasing food demands worldwide, there is a need to maximize food production from the global area of agricultural land. Reclamation of salt-affected soils provides an opportunity for using organic amendments to improve the health of these soils while also sequestering C^18^. Agricultural organic waste and by-products are widely used as soil amendments to increase soil fertility and carbon sequestration. Organic amendments, such as biochar, compost and vermicompost, have potential to minimize the negative impacts of salts on crop production while also providing an opportunity for C sequestration^19,20^. However, application of organic amendments to soils can also increase GHG emissions and contribute to increasing climate change-related problems, especially in salt-affected soils. Numerous studies have reported the GHG emissions form non-saline soils after organic amendments during different crops/cropping systems. For example, Zhang et al.^21^ reported up to 49% more N_2_O emissions when manures were applied during a 30-year wheat-soybean rotation in non-saline soils. Marín-Martínez et al.^22^ reported the similar results with use of cattle manures in vineyards. They reported that N_2_O and CO_2_ emission were increased along with grapes yield and SOC stocks. Win et al.^23^ used compost and cow dung to assess GHG emission in paddy fields in non-saline soils. They reported that compost application resulted in more GHG emissions than cow dung. Romero et al.^24^ did an incubation study using non-saline soil and compared cattle manure and biochar and reported up to 3.80 and 1.80 times more CO_2_ and N_2_O emissions when biochar and manures were applied in combination. Abbas et al.^25^ reported that up to 54%, 90%, 53% and 21% increase in plant height, chlorophyll content, water use efficiency and 1000-grain weight was observed after compost and biochar addition. Dawar et al.^26^ reported that biochar addition during wheat cultivation cause increase in yield and reduced N_2_O emission by 35%. Similarly, vermicompost also showed similar responses for enhancing crop yield by 5% and decreasing GHG emissions^27^. There are few references about the effects of organic substances on GHG emissions under winter wheat cropping on salty soils. Additionally, there is also no study which compares the effects of applied organic amendments on GHG emissions in saline and non-saline soils during wheat crop.

Therefore, here we present the findings from a pot experiment that directly compared the impacts of organic amendments application on (a) greenhouse gas emissions and SOC stocks under wheat cultivation, (b) effects of applied treatments on GWP at different wheat growth stages based on Feekes scale, and (c) crop growth and productivity responses in non-saline and salt-affected soils after treatments application.

## Results

### Pre-analysis of soil properties

Before the start of the experiment, experimental soils were subjected to physico-chemical check. The results obtained after the analysis are presented in Table 1.

**Table 1:** Pedological characteristics of experimental soil.

Parameter	Unit	Non-saline soil	Salt-affected soil
EC_e_	dS m^−1^	1.73 ± 0.04	9.26 ± 0.76
TSS	mmol_c_ L^−1^	17.3 ± 1.35	92.6 ± 2.56
Bulk density	g cm^−3^	1.71 ± 0.02	1.58 ± 0.02
SOC	%	0.73 ± 0.01	0.21 ± 0.01
MBC	mg kg^−1^	324.0 ± 12.3	83.0 ± 1.68
Extractable K	mg kg^−1^	224.0 ± 10.3	97.0 ± 2.42
Available P	mg kg^−1^	6.30 ± 0.57	2.14 ± 0.12
pH	-	7.60 ± 0.55	8.63 ± 0.11
CO_3_^2−^	mmol_c_ L^−1^	Absent	Absent
HCO_3_^−^	mmol_c_ L^−1^	4.0 ± 0.04	7.0 ± 0.17
Cl^−^	mmol_c_ L^−1^	4.1 ± 0.03	54.0 ± 1.57
SAR	(mmol_c_ L^−1^)^1/2^	4.94 ± 0.11	39.0 ± 2.45
Saturation	%	29.18 ± 2.44	30.0 ± 2.25
Texture	-	Sandy loam	Sandy loam
Ca + Mg	mmol_c_ L^−1^	3.1 ± 1.22	4.44 ± 2.57
Na^+^	mmol_c_ L^−1^	6.15 ± 0.91	58.11 ± 1.11

Values given are average of 3 replicates (n = 3). Values after ± sign indicate standard error.

*EC*_*e*_ electrical conductivity, *TSS* total soluble salts, *SOC* soil organic carbon, *MBC* microbial biomass carbon, *K* potassium, *P* phosphorus, *CO*_*3*_^*2−*^ carbonates, *HCO*_*3*_^*−*^ bicarbonates, *Cl*^*−*^ chlorides, *SAR* sodium adsorption ratio.

### Plant growth and yield attributes

All the treatments showed significant differences (p ≤ 0.05) from each other. Table 2 represents the seed germination. It can be seen that seed germination was maximum (100%) in vermicompost and compost-amended soils compared to both control and biochar application in non-saline soil, while 90% in both compost and vermicompost-amended soil 80% in their control and biochar-amended soil. Plant height recorded a maximum 21% increase in vermicompost-amended non-saline soil, while the same treatment also performed best in salt-affected soil with 18% more plant height than its control. No. of spikes per pot were 75% more in vermicompost-amended soil compared to control in non-saline, while 60% more were found in compost-amended soil in salt-affected soil. No. of spikelet were 33% more in non-saline soil, while 42% more in salt-affected soil when compared to their respective controls. Spike length followed the same trend as these were also found maximum in non-saline (40%) and salt-affected soil (29%) more in vermicompost amended soil compared to their controls. Maximum increase in root length was recorded in treatment with vermicompost (28%) in non-saline soil, while 25% increase in both vermicompost and compost amended soil in salt-affected soil. Plant dry biomass production was recorded maximum with 57% increase in non-saline soil with vermicompost, while 56% with the same treatment in salt-affected soil. The maximum increment in grain yield was noted with vermicompost treatment (44%) in non-saline, while 50% increase was obtained with vermicompost over the control in salt-affected soil (Table 2).

**Table 2:** Impacts of applied treatments on different plant growth and yield attributes.

Treatments	Germination percentage (%)		No. of spikelets (plant^−1^)		No. of spikes (pot^−1^)		Plant dry matter (g)		Spikes length (cm)		Root length (cm)		Plant height (cm)		Grain yield (g pot^−1^)	
	Saline	Non saline	Saline	Non saline	Saline	Non saline	Saline	Non saline	Saline	Non saline	Saline	Non saline	Saline	Non saline	Saline	Non saline
Control	80 ± 3a	90 ± 6a	7 ± 0.33d	12 ± 0.34b	5 ± 0.33f.	12 ± 1.16c	18 ± 0.60f.	28 ± 0.16de	7 ± 0.33c	10 ± 0.57e	13 ± 0.57de	18 ± 1.54c	50 ± 1.21f.	67 ± 2.42c	4 ± 0.58e	10 ± 0.13c
Biochar	80 ± 2a	90 ± 8a	10 ± 0.43bc	16 ± 0.32a	8 ± 0.67d	21 ± 0.33a	28 ± 0.16d	44 ± 0.33a	9 ± 0.33a	14 ± 0.16cd	15 ± 0.64d	23 ± 1.46a	60 ± 0.58d	80 ± 3.25b	6 ± 0.88d	18 ± 0.47a
Vermicompost	90 ± 5a	100 ± 3a	7 ± 0.53d	15 ± 0.46a	6 ± 0.58ef	17 ± 0.43bc	26 ± 0.73e	37 ± 0.44c	8 ± 0.16b	12 ± 0.34de	15 ± 0.33d	21 ± 0.84ab	54 ± 3.45e	73 ± 5.21a	5 ± 0.44de	15 ± 0.51b
Compost	90 ± 5a	100 ± 7a	8 ± 0.35cd	16 ± 0.42a	8 ± 0.29de	20 ± 0.33ab	27 ± 0.14de	40 ± 0.29b	9 ± 0.25b	13 ± 0.24cd	12 ± 0.43e	20 ± 0.46bc	56 ± 1.32e	76 ± 13.4b	5 ± 0.56de	16 ± 0.45b
LSD	≤ 0.001		≤ 0.01		≤ 0.001		≤ 0.003		≤ 0.05		≤ 0.05		≤ 0.01		≤ 0.001	

Error bars represent the standard error (n = 3). Different letters indicate statistical difference (LSD, *p* < 0.05).

### Plant physiological attributes

All the applied amendments significantly (p < 0.05) increased the chlorophyll contents in wheat in both non-saline and salt-affected soils when compared with the control. Maximum chlorophyll increment was observed with vermicompost treatment (24%), while 22% increment was recorded in salt-affected soil after the application of the same treatments. Photosynthetic rate was recorded 47% and 53% increase over control with the same treatment application, i.e., vermicompost in both soils, respectively. Stomatal conductance also followed the same pattern with 60% and 12% increase over respective control in non-saline and salt-affected soils, respectively. The increase in relative water contents for non-saline and salt-affected soil treated with vermicompost were noted 38% and 27% over the control (Table 3).

**Table 3:** Impacts of applied treatments on crop physiological attributes.

Treatments	Chlorophyl contents (SPAD)		Stomatal conductance (mol m^−2^ s^−1^)		Relative water contents (%)		Photosynthetic rates (m^−2^ s^−1^)	
	Saline	Non saline	Saline	Non saline	Saline	Non saline	Saline	Non saline
Control	41 ± 0.17e	46 ± 0.56d	0.60 ± 0.01d	0.50 ± .01b	41 ± 0.44e	45 ± 0.20de	2.20 ± 0.12d	2.50 ± 0.11b
Biochar	50 ± 0.61bc	57 ± 1.07a	0.68 ± .01bc	0.78 ± .03a	52 ± 1.55bc	62 ± 2.14a	3.26 ± 0.23bc	3.30 ± 0.14a
Vermicompost	48 ± 0.54cd	54 ± 1.19ab	0.70 ± .02d	0.80 ± .02a	48 ± 0.60de	54 ± 0.22bc	3.00 ± 0.10bc	3.20 ± 0.09b
Compost	46 ± 0.38d	52 ± 1.04bc	0.69 ± .02cd	0.79 ± .03a	48 ± 0.03cd	55 ± 0.58b	3.22 ± 0.31cd	3.21 ± 0.21b
LSD	≤ 0.001		≤ 0.001		≤ 0.001		≤ 0.05	

The values after ± represent the standard error (n = 3). Different letters indicate statistical difference (LSD, *p* < 0.05).

### Soil and plant nutrition acquisition

Same as for the plant growth, yield, and physiological parameters, all the applied amendments significantly (p < 0.05) increased N in wheat plant and soil in both non-saline and salt-affected soils compared with the control. However, the maximum N increase in plant was observed with the vermicompost treatment (62%) in non-saline, and 56% increase in salt-affected soil with the same treatment The total N in soil was found maximum in vermicompost amended soil up to 107% increase in non-saline, while 60% increase each in compost and vermicompost amended soils compared to respective controls (Table 4).

**Table 4:** Impacts of applied treatments on soil and plant nitrogen uptake after wheat harvest.

Treatments	Nitrogen in plant (mg kg^−1^)		Nitrogen in soil (mg kg^−1^)	
	Saline	Non saline	Saline	Non saline
Control	0.39 ± 0.03e	0.66 ± 0.02c	0.20 ± 0.008e	0.38 ± 0.001c
Biochar	0.61 ± 0.003cd	1.08 ± 0.03a	0.32 ± 0.020cd	0.71 ± 0.011a
Vermicompost	0.61 ± 0.001cd	1.05 ± 0.005ab	0.32 ± 0.032cd	0.65 ± 0.006ab
Compost	0.55 ± 0.001d	0.97 ± 0.005b	0.26 ± 0.002de	0.59 ± 0.009b
LSD	≤ 0.05		≤ 0.05	

Error bars represent the standard error (n = 3). Different letters indicate statistical difference (LSD, *p* < 0.05).

### Post-harvest soil analysis

All the applied amendments showed a positive effect on soil pH_s_, EC, and other ionic and nutritional characteristics in both non-saline and salt-affected soils after wheat growth. Applied organic amendments showed a minor but positive effect on soil pH. In non-saline soil, amendments have a minor decrease in soil PH_,_ i.e., up to 3.30%, maximum decrease with the application of vermicompost over the control. While in salt-affected soil, a maximum decrease of 16% was observed with vermicompost compared to the control (Table 5). In non-saline soil, a minor decrease in EC was observed, i.e., 1% with both compost and vermicompost over the control, while 7% decrease was observed in salt-affected soil with same treatment. The SOC recorded maximum with vermicompost treatment up to 25 and 67% increase in non-saline and salt-affected soil, respectively with vermicompost. In soil MBC, 10 and 23% increase was recorded in non-saline and salt-affected soil, respectively with the application of vermicompost in both soils (see Table 5).

**Table 5:** Post-harvest characteristics of experimental soil.

Parameter	Unit	Non-saline soil	Salt-affected soil
EC_e_	dS m^−1^	2.53 ± 0.12	8.37 ± 0.21
TSS	mmol_c_ L^−1^	15.60 ± 1.17	41.32 ± 2.45
SOC	%	0.91 ± 0.08	0.35 ± 0.05
MBC	mg kg^−1^	355.0 ± 11.3	102.0 ± 4.53
Extractable K	mg kg^−1^	345.0 ± 21.2	143.0 ± 9.24
Available P	mg kg^−1^	11.34 ± 1.05	5.11 ± 0.23
pH_s_	-	7.35 ± 0.34	8.03 ± 0.09
SAR	(mmol_c_ L^−1^)^1/2^	9.34 ± 0.95	21.0 ± 1.13

Values given are average of 3 replicates (n = 3). Values after ± sign indicate standard error.

*TSS* total soluble salts, *SOC* soil organic carbon, *MBC* microbial biomass carbon, *SAR* sodium adsorption ratio.

### Post-harvest SOC stocks under applied treatments

**Figure 1:**
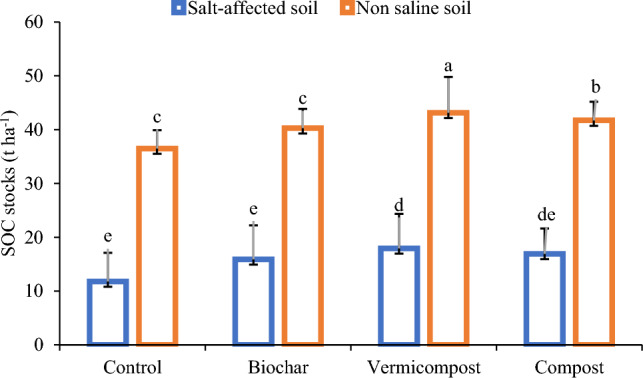
Effect of applied treatments on soil organic carbon stocks in saline and non saline soils after wheat harvesting. Error bars represent standard error. The data presented is average of three replications (n = 3), while error bars present standard errors.

The calculated SOC stocks at the end of the experiment are presented in Fig. 1. All the treatment except control enhanced the SOC stocks both type of soils, but the maximum increase was noted in vermicompost amended saline soil (17.96 t ha^−1^) compared to 11.80 t ha^−1^ in its control. The increase in SOC stocks in non-saline soil was also recorded in the same treatment which was 43.13 t ha^−1^ compared to 36.50 t ha^−1^ in its control (Fig. 1).

### Greenhouse gas emissions

**Figure 2:**
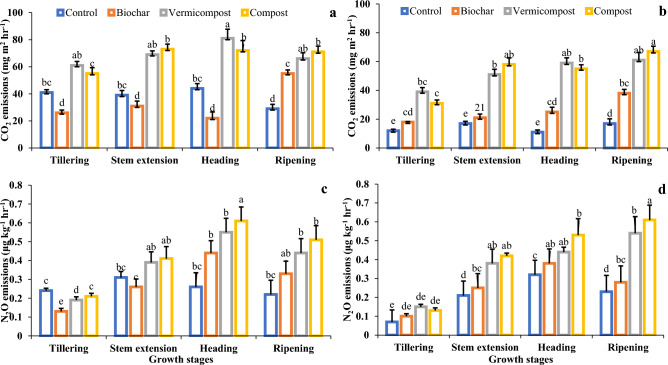
Carbon dioxide (**a**) non-saline, (**b**) salt-affected soil), and nitrous oxide (**c**) non-saline, (**d**) salt-affected soil) gas emissions during different stages of wheat plant. The data presented is average of three replications (n = 3), while letters are showing statistical significance at *p* < 0.05.

There were significant differences between the carbon and nitrous oxide emissions from non-saline and salt-affected soils (Fig. 2). Treatment with biochar application proved the most effective in restricting the carbon and nitrogen emissions and exhibited the least carbon emissions, i.e., 25% in non-saline soil followed by 60% and 54% in treatments with vermicompost and compost (Fig. 2). Similarly, biochar application also caused 11% of carbon emissions, followed by 16% and 38% in vermicompost and compost-amended salt-affected soil (Fig. 2a,b). Same as the case, biochar treatment also controlled maximum nitrogen (N_2_O) emissions compared to vermicompost and compost treatment. In non-saline soil, biochar treatment caused 0.87% less N emissions compared to 0.81% and 0.79% emissions in vermicompost- and compost-amended soil. In salt-affected soil, a similar trend was recorded where biochar caused 0.90% fewer N_2_O emissions compared to 0.85% and 0.87% in vermicompost and compost-amended salt-affected soil (Fig. 2c,d).

### Global warming potential

Table 6 provides the estimated total global warming potential (GWP) in the non-saline and salt-affected soils applied with the organic amendments (i.e., biochar, vermicompost, and compost). In non-saline soil, the biochar treatment considerably reduced the GWP even compared to the control, whereas 77% and 81% higher GWP is estimated with treatment compost and vermicompost in comparison to the control. In the case of salt-affected soil, the application of biochar raised the GWP in comparison to the control, whereas the GWP of compost and vermicompost application was 2 times higher than the biochar amendment.

**Table 6:** Global warming potential (kg CO_2_-equivalents ha^−1^) in non-saline and salt-affected soils.

Treatments	Non-saline soils	Salt-affected soils
Control	306.61	114.49
Biochar	268.69 (− 12.37%)	204.60 (+ 78.7%)
Vermicompost	554.93 (+ 81%)	420.89 (+ 267.62%)
Compost	543.03 (+ 77%)	423.00 (+ 267.46%)

Characterization factor for a 100-year time frame is 296 and 1 for N_2_O and CO_2_, respectively (IPCC 2001). Values in parentheses representing the percent increase (+) or decrease (−).

### Correlation and principal component analysis

#### Correlation among the parameters

**Figure 3:**
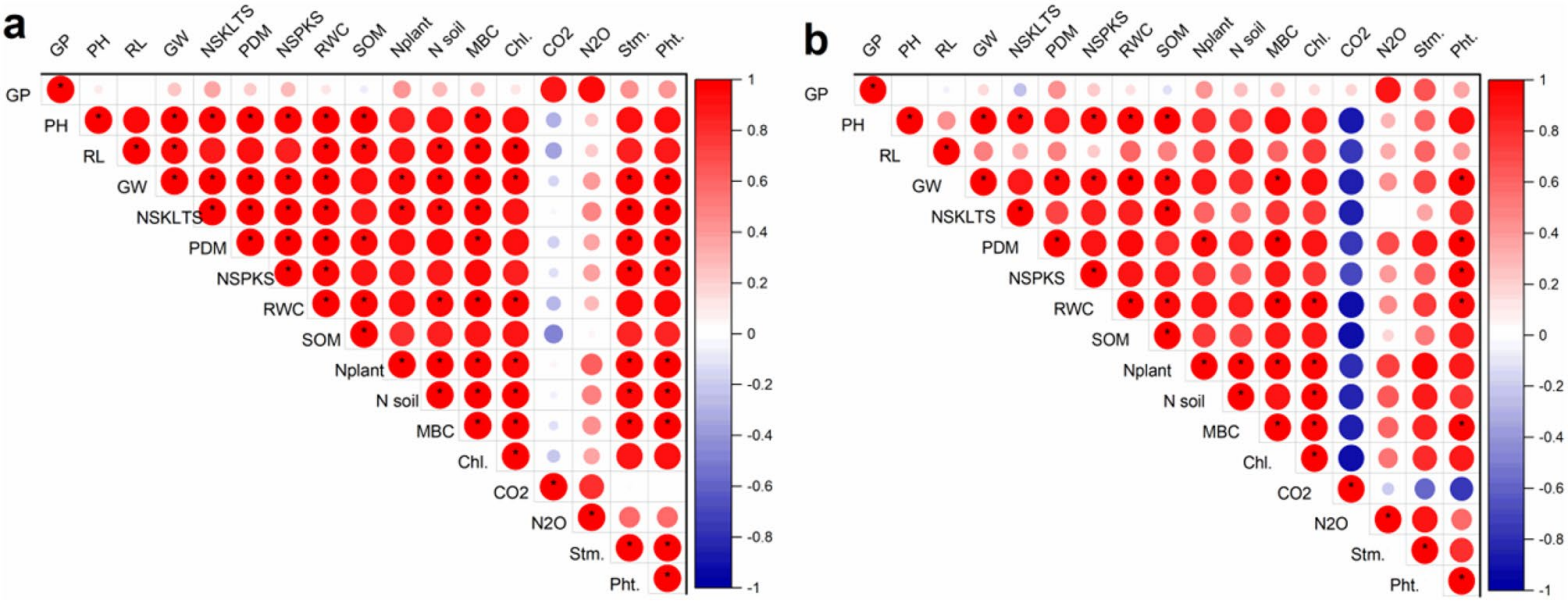
Pearson correlation between the studied parameters in (**a**) non-saline, and (**b**) salt-affected soils. *GP* germination percentage, *PH* plant height, *RL* root length, *GW* grain weight, *NSKLTS* no. of spikelet, *PDM* plant dry matter, *NSPKS* no. of spikes, *RWC* relative water contents, *SOM* soil organic matter, *Nplant* nitrogen in plant, *Nsoil* nitrogen in soil, *MBC* microbial biomass carbon, *Chl.* chlorophyl contents, *CO*_*2*_ carbon dioxide emissions, *N*_*2*_*O* nitrous oxide emissions, *Stm.* stomatal conductance, *Pht.* photosynthetic rate.

Pearson’s correlation coefficient was calculated to quantify relationships between various parameters of the study. Figure 3a shows Pearson’s correlations and levels of significance for the relationship between the plant height, grain weight, yield, plant dry matter production, soil organic matter, no. of spikes, microbial biomass carbon, and relative water contents in non-saline soil. The nitrogen emissions were positively correlated with nitrogen in plant and soil and microbial biomass carbon. The same is presented about the salt-affected soil in Fig. 3b, where plant height showed a positive relationship with grain yield, no. of spikes, dry matter, and microbial biomass carbon (Fig. 3b).

#### Principal component analysis (PCA)

**Figure 4:**
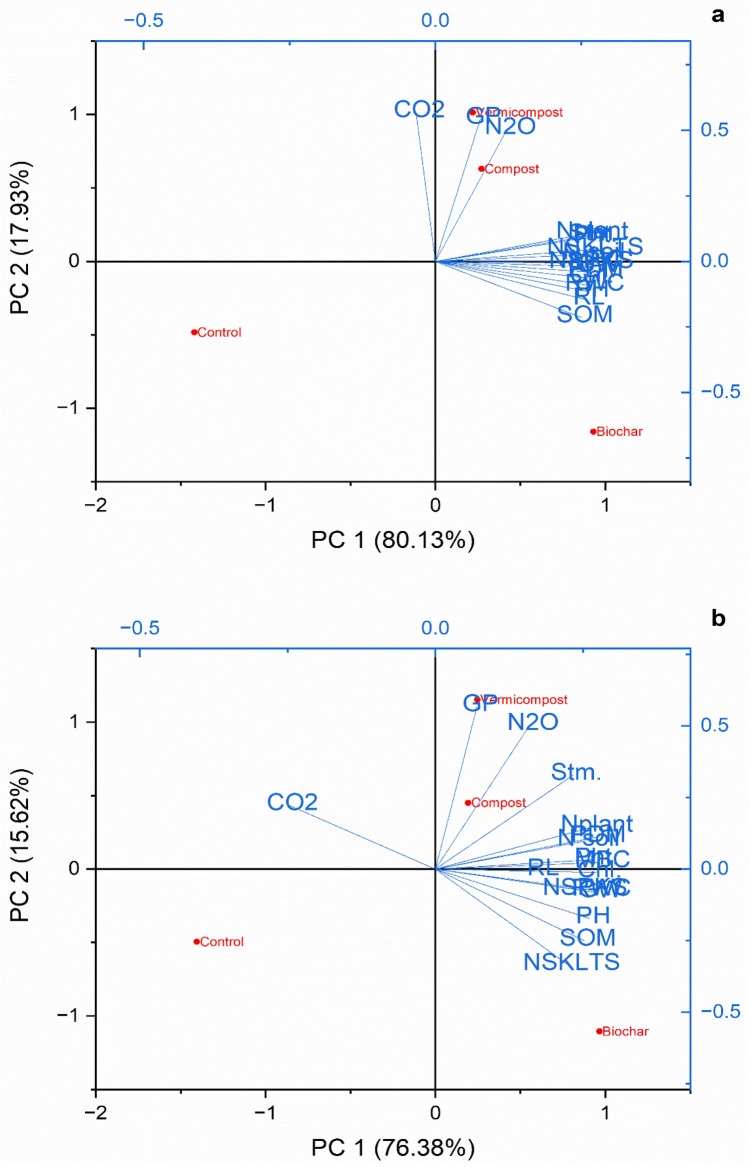
Principal component analysis explaining the effects of applied treatments on (**a**) non saline, and (**b**) salt-affected soil.

The PCA analysis shows that apart from the germination percentage and greenhouse gas emissions, all the remaining components of the study (i.e., growth, yield and physiological attributes, and soil properties) accumulated 80.1% of the total variance (Fig. 4) in non-saline soil. The first principal component (PC1) explained 80.1% of the variance and reflected the positive coordination with the parameters. The second principal component (PC2) explained the 17.9% covariation of all increased factors. The points near the lines originating from the center depicted higher values as compared to distant points (Fig. 4a). In the salt-affected soil, PC1 showed 76.4% variance, while PC2 showed 15.6% variance. The same coordination pattern was also noted in the salt-affected soil as seen in non-saline soil (Fig. 4b).

## Discussion

### Crop growth and yield parameters

The application of organic amendments significantly improves soil health attributes such as SOC, soil pH, moisture, and productivity in terms of crop yield^28^. In our study, vermicompost amendment produced the best results in terms of seed germination, plant growth, and yield attributes. Plant height, dry matter production, spikes number, length, etc., were found more in vermicompost-amended non-saline soil, compared to salt-affected soil with the same treatment. This could be due to the enrichment of both soils with readily available plant nutrients, which helps in better plant growth. Aslam et al.^29^ reported that vermicompost application significantly improved plant growth and yield up to 5.37 t ha^−1^. Due to large particulate surface areas, composts provide many micro sites for microbial activity and the strong retention of nutrients. Vermicompost contains most nutrients in plant-available forms that ultimately enhance the biochemical, yield, and quality attributes of the crops^30^. Some growth-improving products, such as hormones, humates, and amino acids, are also produced as a by-product of microbial and earthworm activity^31^. These properties of vermicompost might be the reason for the improvement of the quality of plant growth, yield, and soil health, as we have observed in our study^32^. Thus, the increasing effect of vermicompost on soil nutrient availability leads to an increase in plant mineral nutrition.

In the present experiment, plant height, number of tillers, spike length, number of spikelet per spike were significantly increased by using compost. This might be due to enhanced nutrient use efficiency by the slow release of nutrients and reducing their losses in the case of compost application^33^. Previously the influential role of compost in improving plant height, number of tillers, spike length, and number of spikelet per plant was also reported by Ibrahim et al.^34^. In salt-affected soils, compost increases crop production by improving soil biological and physical, and chemical properties and alleviates salt stress by neutralizing soil reaction^35^. Vermicompost also considerably increased plant height, fresh weight, and dry weight. While the effect of vermicompost compared with other treatments for root length, fresh root weight, and dry root weight remained non-significant. Befrozfar et al.^36^ concluded that vermicompost application improves the growth parameters such as dry matter production, leaf area, and yield in non-saline and salt-affected soils. The growth of plants varied with the vermicompost treatments because growth-promoting substances are released at different rates in different treatments; this might be due to the amount of nutrient content and microbes promoting the plant growth^37^.

### Plant physiological attributes

Organic amendments also showed positive impacts on the chlorophyll contents of plants in non-saline and salt-affected soils. Similar to the findings of this experiment, Abd El-Mageed et al.^38^ also observed that biochar application improved stomatal conductance, relative chlorophyll contents (SPAD value), plant production, and photosynthetic efficiency. The properties of biochar, like high water-holding capacity and porous structure, maintain sufficient moisture in the soil that plant absorbs easily, and ultimately, plant shows an adequate level of relative water contents (RWC)^39^. Akhtar et al.^40^ also observed improved plant water status with biochar application. The beneficial results with vermicompost and compost regarding gases exchange attributes in both non-saline and salt-affected soils were observed due to an increase in N contents with the application of these amendments that accelerated plant development and the leaf area index, which in turn increased light absorption. Therefore, when vermicompost and compost were applied, the chlorophyll contents of leaves increased^37^. The improvement in RWC with the application of compost and vermicompost in non-saline and salt-affected soils could be justified by balancing between ion absorption and water loss. Compost and vermicompost play a significant role in an increase in RWC and N uptake, which is essential for photosynthesis and encourages plant growth and development^41^.

### Post-harvest changes in soil physico-chemical, biological characteristics and soil organic carbon turn over

It was observed that the compost and vermicompost amendments significantly improved the physio-chemical parameters of soil after harvesting of wheat crop. Previously, Antonangelo et al.^42^ also reported maximum yields and availability of nutrients in soil after harvest in a treatment where compost and/or vermicompost was supplied. Similarly, Ros et al.^43^ and Pratibha et al.^44^ also found that the application of vermicompost in soils significantly improved the post-harvest physio-chemical attributes of the soil. Organic amendments significantly decreased EC and pH of salt-affected soils (Qadir et al. 2022). The application of compost and biochar improved the physical conditions of the soil, which increased the leaching ability of soil and thus resulted in a considerable reduction in salts in the root zone^45^. Biochar is claimed to achieve several sustainability goals, including C sequestration, soil health, and plant growth improvements^18,46^. In this study, addition of vermicompost and compost showed good results for increasing SOC in both non-saline and salt-affected soils. Nutrients richness and higher potential to supply nutrients to soils and plants increases SOC content, microbial activity such as nutrient cycling and nitrogen fixation, and soil fertility^47^. The application of compost and vermicompost increased more SOC in both soils compared to control and biochar, as biochar releases slow carbon and resists fast decomposition when added into soils. Soil organic carbon is a sink of atmospheric CO_2_, thereby counteracting the mechanism of global warming^48^. In this experiment, the higher rates of amendments application were responsible for the significant increase in SOC accumulation soils. In case of salt-affected soil, the soil was previously un-managed. The addition of biochar, vermicompost and compost increased soil aggregation by acceleration to form microaggregates and macroaggregates, which are then cemented or coagulated together^18^. In our investigation, the addition of organic amendments probably boosted the development of organic soil colloids, which encouraged the creation of organo-mineral complexes and caused aggregates to form and subsequent increase in SOC storage. The availability of nutrients was also boosted by the organic additions and decomposition.

### Nitrogen in plant and soil

In vermicompost treatment, relatively higher N contents were recorded in both salt-affected and non-saline soil, which might be due to increased N as nitrogenous excretory products of earthworms in the vermicompost^49,50^. Earthworms were reported to have a great impact on nitrogen transformation in vermicompost formation, and due to increased N mineralization, the major portion of the N retained in soil as the plant available form, i.e., nitrate and thus adequate N uptake by the plant when vermicompost was applied to soil^49,51^. The compost treatment also depicted an increase in N content as compared to the control, which may be due to the high mineral N content of compost and lower dry matter content.

### Greenhouse gas emissions and global warming potential

The present study shows that biochar application is the most effective in restricting carbon and nitrogen emissions from soil and offsetting global warming potential (GWP) because of its higher ability to sequester C in the soil^18^. It is observed that the gas emissions in non-saline soil are considerably higher than the degraded soils. This may be due to higher microbial activity in non-saline soil compared to salt-affected soils. Application of organic amendments to soil can increase GHG emissions and can alleviate GWP^52^, even though they improve crop productivity and net carbon budget of the soils^53^. Vermicompost and compost increased the microbial and enzymatic activity in soil, which increased the mineralization rate of organic matter in the soil^31^ and thus resulted in more CO_2_ production^52^. This provides the reason for increased CO_2_ emission and estimated total GWP with vermicompost and compost application. However, the application of the same quantity (on a C equivalent basis) of biochar has resulted in enhanced soil carbon sequestration with restricted GHG emissions.

## Conclusions

In this study, the impact of various soil organic amendments on GHG emissions, along with plant growth and yield parameters, was determined comparatively in non-saline and salt-affected soils. The results showed that soil organic carbon increased over the control by 18% in the non-saline soil and 52% in the salt-affected soil. The grain yield increase was recorded by 73% and 53% in non-saline and the salt-affected soil, respectively. The addition of vermicompost caused maximum carbon emission which were 46% in the non-saline soil and 74% in the salt-affected soil, compared to control, while 57 and 62% more nitrous oxide emissions with same treatments in the non saline and salt-affected soil, respectively. The global warming potential was recorded 267 and 81% more compared to control in saline and non-saline soils treated with vermicompost, respectively but biochar treated soil emitted 12% less GHGs in the non-saline soil. The results of this study can be recommended for different crops as organic amendments like biochar an economic and environmental point of view to reduce the use of chemical fertilizers and enhance SOC pool with restricted GHG emissions.

## Materials and methods

A pot trial was conducted during the winter season of 2021–2022 (15 Nov–6 April) in the glass house (31.2600 N, 73.0419 E) at the Institute of Soil and Environmental Sciences (ISES), University of Agriculture Faisalabad (UAF). Bulk soil samples (depth 0–30 cm) were collected from the salt-affected area of Dijkot (31°11′25″ N 73°03′55″ E), District Faisalabad, Pakistan, while non-saline soil was collected from the farm area of ISES (31°26′20″ N 73°04′11″ E), UAF. These soils belong to aridisol class having clay loam texture and developed under arid condition through alluvium derived from Himalayas. Before analysis, the soil was air-dried, sieved using a 2-mm sieve, and processed further for the determination of physico-chemical and biological characteristics as detailed below.

### Determination of soil physico-chemical and biological properties

Soil pH was determined using the pH meter (Hanna HI-83141), while electrical conductivity (EC) was measured using a pre-calibrated EC meter (Lovibond SensoDirect con200), while soil texture was determined using hydrometer through Bouyoucos method^54^. Soil organic matter and nutrient contents were quantified using standard analytical procedures i.e., soil organic matter was determined by the Walkley Black method^55^, while SOC was determined by multiplying SOM to 0.58. Total soil nitrogen was determined using the Kjeldahl apparatus by a two-step digestion and distillation process^56^. Available phosphorus was determined using the Olsen method^57^. Extractable potassium was determined using the method devised by Norman^58^. Microbial biomass carbon (MBC) was determined by fumigation-extraction method^59^. The total soluble salts (TSS), residual sodium carbonates (CO_3_^2−^ and HCO_3_^−^), and Cl^−^ were measured using methods outlined by the US Salinity Laboratory Staff (1954)^60^. The sodium adsorption ration (SAR) was determined using the following equation;

$${r}_{SAR }=\frac{{C}_{Na}}{\frac{\sqrt{{C}_{Na}+{C}_{Mg}}}{2}}$$

where ${{\text{r}}}_{{\text{SAR}}}$ is the SAR (mmol dm^−3^)^1/2^), ${{\text{C}}}_{Na}$ is the concentration of sodium ions (mmol_c_ L^−1^) and ${{\text{C}}}_{Mg}$ is concentration of magnesium ions (mmol_c_ L^−1^).

### Climatic data

**Figure 5:**
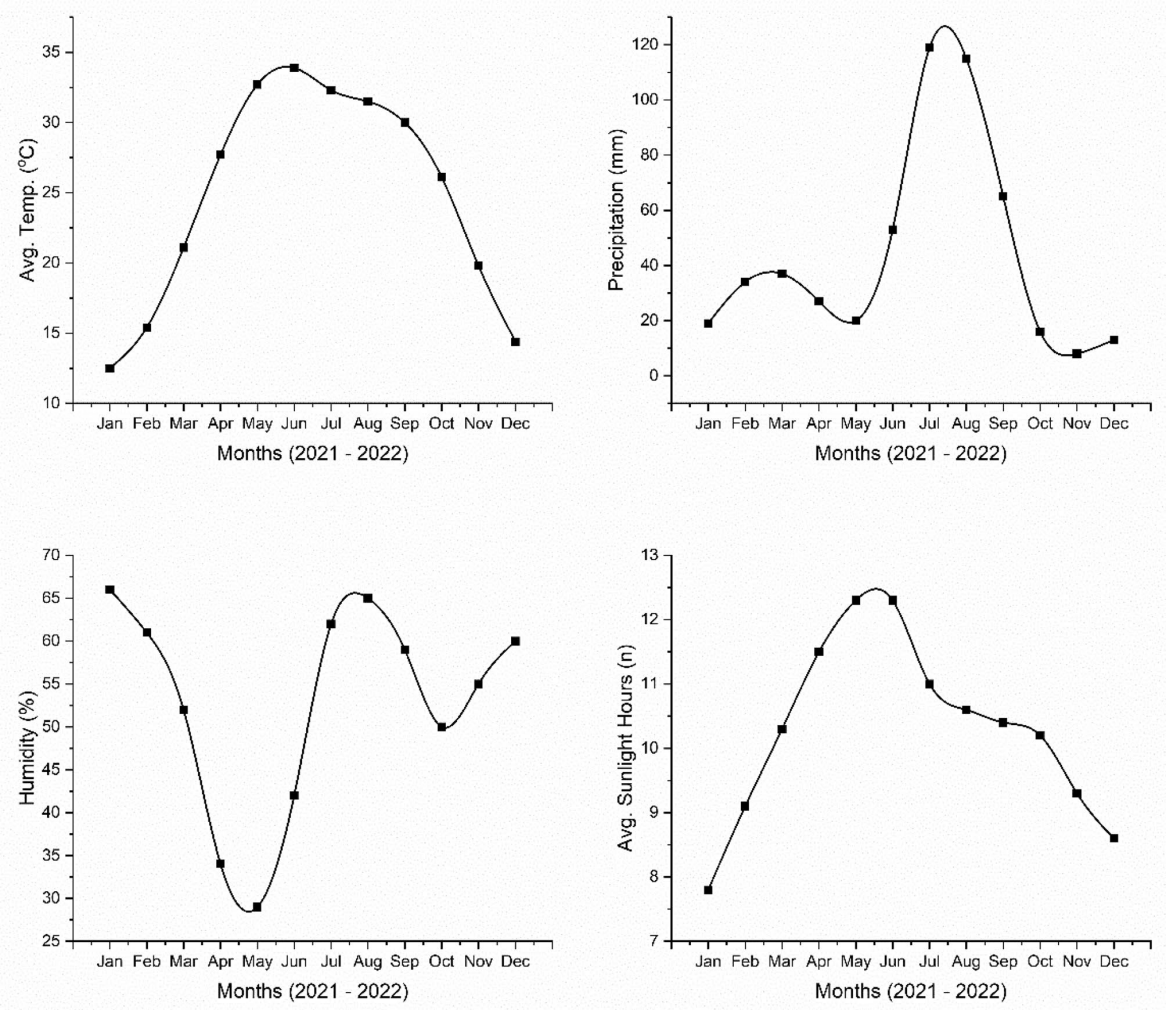
Meteorological parameters recorded during the study span.

The weather data conditions prevailing in the experimental year 2021–2022 were obtained from the meteorological observatory, ISES, UAF located near at 31.2621 N and 73.0419 E (Fig. 5). As the Faisalabad lies in arid to semi-arid region based on the climate, the majority of the study span had continuous sunlight and less temperature variations from the averages of both winter and summer seasons. The annual maximum and minimum temperature were observed as 45.5 °C and 19.1 °C respectively, while the yearly precipitation ranged from 300 to 400 mm.

### Experimental design and crop husbandry

The experiment was divided into two sets, each having four treatments. The first set was comprised of non-saline soil, while the second set was salt-affected soil. Both sets had the same treatments, i.e., control (un-amended), biochar, vermicompost and compost amendments. Treatments were applied at the rate of 1% of their organic carbon equivalent basis, i.e., 4.05 t ha^−1^ biochar, 11.80 t ha^−1^ vermicompost, and 9.15 t ha^−1^ compost. The pots had a capacity of 10 kg with diameter and depth of 25 cm were filled with (a) non-saline soil and (b) salt-affected soil. There were three treatments + control (per soil type). All these treatments were replicated three times and arranged in a completely randomized design in the glass house under natural conditions. Each pot was seeded with 8–10 seeds of wheat (*Triticum aestivum* L.) of the Akbar 2019 variety. After one week of seed germination, thinning was done by keeping 5 plants per pot, maximally spaced. Recommended doses of NPK were applied in all treatments using urea, (di-ammonium phosphate) (DAP), and sulfate of potash (SOP); applied N was 79 kg ha^−1^, applied P was 57 kg ha^−1^ and applied K was 62 kg ha^−1^. Canal water was used for irrigation and each irrigation was applied after 15 days of interval, while fertilizers were applied in 3 splits (at sowing, after 1st irrigation, and 2nd irrigation. The physico-chemical composition of the canal water and applied organic amendments are given in Tables 7 and 8, respectively.

**Table 7:** Properties of canal water used for irrigation during the study.

Properties	Values	Units
pH	8.21 ± 0.53	–
EC	0.93 ± 0.03	dS m^−1^
SAR	1.56 ± 0.07	(mmol_c_ L^−1^)^1/2^
RSC	1.33 ± 0.01	mmol_c_ L^−1^
CO_3_^−^	–	–
HCO_3_^−^	3.37 ± 0.53	mmol_c_ L^−1^
Cl^−^	0.76 ± 0.01	mmol_c_ L^−1^
Ca + Mg	2.04 ± 0.11	mmol_c_ L^−1^
Na^+^	1.58 ± 0.01	mmol_c_ L^−1^

Values after ± represent standard error (n = 3).

*EC* electrical conductivity of the saturated soil paste extract, *SAR* sodium adsorption ratio, *RSC* residual sodium carbonates.

**Table 8:** Properties of organic amendments used in the study.

Properties	Units	Biochar	Compost	Vermicompost
pH	–	7.65 ± 0.33	6.88 ± 0.46	7.23 ± 0.53
EC	dS m^−1^	2.59 ± 0.05	1.57 ± 0.02	5.93 ± 0.03
OC	%	67.0 ± 3.24	37.60 ± 2.21	29.43 ± 3.56
CEC	cmol (+) kg^−1^	15.75 ± 1.35	–	–

Values given are average of 3 replicates (n = 3). Values after ± sign indicate standard error.

*EC* electrical conductivity, *OC* organic carbon, *CEC* cation exchange capacity.

### Determination of plant growth, yield, and physiological attributes

At the vegetative growth stage (40 days after germination), chlorophyll content was measured using a SPAD 200 chlorophyll meter from top fully developed leaves (Hussain et al. 2000a). The relative water content (RWC) was measured in a fresh leaf cut from each pot following the method of Mayank et al. (2004); the fresh weight, ${M}_{{\text{fresh}}}$ (g), was measured before keeping the leaf at 100% humidity at 40 °C for 48 h to provide the turgid fresh weight, ${M}_{{\text{turgid}}}$ (g), and finally drying it in an oven to constant weight to give the dry weight, ${M}_{{\text{dry}}}$ (g). The following formula was used to calculate relative water content, ${P}_{{\text{RWC}}}$ (%):

$${P}_{{\text{RWC}}}=\frac{{M}_{{\text{fresh}}}-{M}_{{\text{dry}}}}{{M}_{{\text{turgid}}}-{M}_{{\text{dry}}}}\times 100$$

At plant maturity stage (142 days after germination), plant growth and yield attributes (plant height, fresh and dry weights, number of tillers, spikes length, root length, no. of spikes, spikes length, and grain yield) were measured. Plant height, root length, and spikes lengths of all the plants in a pot were measured using measuring tape having accuracy ± 1.10 mm and average was calculated and reported. To measure root length, the plants were well-watered, then gently extracted from pots and carefully shaken off to remove excess soil. Fresh and dry weights were measured using top loading balance having accuracy ± 0.001 g.

### Assessment of greenhouse gas emissions

The greenhouse gas emissions in wheat crop were measured 4 times according to the growth stages in the Feekes scale (tillering (30 days), stem extension (50 days), heading (60 days), and ripening (120 days)). For N_2_O emissions, the static chambers were used and emissions were recorded and analyzed as described by Bolan et al.^61^, while soil respiration or CO_2_ emissions were measured using the method described by Anderson^62^ with the following equation;

$${R}_{{\text{soil}}}=\frac{\left(12\times {V}_{1}\times 0.1\right)\times \left({V}_{{\text{B}}}-{V}_{3}\right)}{2\times {V}_{2}}$$

where ${R}_{{\text{soil}}}$ the soil respiration (mg m^2^ h^−1^), ${V}_{1}$ is the volume (cm^3^) of NaOH used for CO_2_ trapping, ${V}_{2}$ is the volume (cm^3^) of NaOH used for titration, ${V}_{{\text{B}}}$ is blank sample reading (cm^3^), and ${V}_{3}$ is the volume (cm^3^) of HCl used for titration, and 2 is the conversion factor, 12 is the molecular weight of C, while 0.1 is the molarity of HCl used for titration. As 1 ml 0.1 M HCl is equivalent to 2 mg CO_2_ emitted, it is used as a conversion factor.

### Global warming potential calculation

The global warming potential (GWP) was calculated by multiplying the carbon dioxide equivalent of a quantity of C and N gas by the mass of the gas. For this purpose, the amount of carbon dioxide was 1, while the amount of nitrous oxide was multiplied by 298 to make it 1 tone of CO_2_ equivalents^63^.

### Statistical analysis

The obtained data was statistically analyzed by R package using R-studio (v4.2.0). Figures and correlation plots were developed using OriginPro 2022b (Origin Lab Corp., USA). Analysis of variance and the Tukey HSD test was used to assess the significance of difference between the treatments.

### Statement of ethical standards

The Study complies with local and national guidelines and regulations.

## Data Availability

All data generated or analyzed during this study are included in this article. However, raw data of this manuscript can be provided by corresponding author on reasonable request. Besides, plant material data used is followed all the legislation. There is no conflict of interest in using plant material and data.
