# Author Correction: Greenhouse gas emissions, carbon stocks and wheat productivity following biochar, compost and vermicompost amendments: comparison of non-saline and salt-affected soils

**DOI:** 10.1038/s41598-024-68305-x

**Published:** 2024-08-06

**Authors:** Zia Ur Rahman Farooqi, Ayesha Abdul Qadir, Sehrish Khalid, Ghulam Murtaza, Muhammad Nadeem Ashraf, Shafeeq-ur-Rahman, Wasim Javed, Muhammad Ahmed Waqas, Minggang Xu

**Affiliations:** 1 Institute of Soil and Environmental Sciences, University of Agriculture, Faisalabad, 38040 Pakistan; 2 Water Science and Environmental Engineering Research Center, College of Chemical and Environmental Engineering, Shenzhen University, Shenzhen, 518060 China; 3 MOE Laboratory for Earth Surface Processes, College of Urban and Environmental Sciences, Peking University, Beijing, 100871 China; 4 Punjab Bioenergy Institute, University of Agriculture, Faisalabad, 38040 Pakistan; 5 Department of Agroecology, Aarhus University, Blichers Alle 20, PO BOX 50, 8830 Tjele, Denmark; 6 Institute of Eco-Environment and Industrial Technology, Shanxi Agricultural University, Shanxi Province Key Laboratory of Soil Environment and Nutrient Resources, Taiyuan, 030031 China

Correction to: *Scientific Reports* 10.1038/s41598-024-56381-y, published online 02 April 2024

The original version of this Article contained errors in Table 1 and Table 5.

Table 1 contained errors in the values given for “ECe” and “pH” in the column “Unit”. The correct and incorrect values appear below.

Incorrect:

**Table:** 

Parameter	Unit
EC_e_	mmol_c_ L^−1^
pH	dS m^−1^

Correct:

**Table:** 

Parameter	Unit
EC_e_	dS m^−1^
pH	-

Additionally, Table 5 contained errors in the values given for “EC_e_” and “pHs” in the column “Unit”. The correct and incorrect values appear below.

Incorrect:

**Table:** 

Parameter	Unit
EC_e_	mmol_c_ L^−1^
pH_s_	dS m^−1^

Correct:

**Table:** 

Parameter	Unit
EC_e_	dS m^−1^
pH_s_	-

The original Article has been corrected.

